# Is the oxidative potential of components of fine particulate matter surface-mediated?

**DOI:** 10.1007/s11356-022-24897-3

**Published:** 2022-12-23

**Authors:** Karsten Baumann, Marco Wietzoreck, Pourya Shahpoury, Alexander Filippi, Stefanie Hildmann, Steven Lelieveld, Thomas Berkemeier, Haijie Tong, Ulrich Pöschl, Gerhard Lammel

**Affiliations:** 1 Multiphase Chemistry Department, Max Planck Institute for Chemistry, Mainz, Germany; 2 Department of Environmental Sciences and Engineering, University of North Carolina, Chapel Hill, USA; 3 Picarro Inc, Santa Clara, USA; 4 Air Quality Processes Research Section, Environment and Climate Change Canada, Toronto, Canada; 5 Chemistry Department, Trent University, Peterborough, Canada; 6 Institute of Surface Science, Helmholtz-Zentrum Hereon, Geesthacht, Germany; 7 Research Centre for Toxic Compounds in the Environment, Masaryk University, Brno, Czech Republic

**Keywords:** Aerosol, Inhalation, Toxic quinones, Alveolar surfactant, Acellular assay

## Abstract

Redox-active substances in fine particulate matter (PM) contribute to inhalation health risks through their potential to generate reactive oxygen species in epithelial lung lining fluid (ELF). The ELF’s air–liquid interface (ALI) can play an important role in the phase transfer and multi-phase reactions of redox-active PM constituents. We investigated the influence of interfacial processes and properties by scrubbing of coated nano-particles with simulated ELF in a nebulizing mist chamber. Weakly water-soluble redox-active organics abundant in ambient fine PM were reproducibly loaded into ELF via ALI mixing. The resulting oxidative potential (OP) of selected quinones and other PAH derivatives were found to exceed the OP resulting from bulk mixing of the same amounts of redox-active substances and ELF. Our results indicate that the OP of PM components depends not only on the PM substance properties but also on the ELF interface properties and uptake mechanisms. OP measurements based on bulk mixing of phases may not represent the effective OP in the human lung.

**Supplementary Information:**

The online version contains supplementary material available at 10.1007/s11356-022-24897-3.

## Introduction

Fine particulate matter (PM_2.5_) is believed to contribute 80% to the total excess mortality of ambient air pollution, which causes millions of excess deaths per year (COMEAP 2018; Burnett et al. 2018; Lelieveld et al. 2020). PM_2.5_ contains redox-active species, i.e. transition metal ions, such as copper and iron, and oxidized polycyclic aromatic compounds, such as quinones (O_2_PAH). Quinones and also other nitrated and oxygenated polycyclic aromatic compounds (PACs) are toxic (Andersson et al. 2009; Park & Park 2009; Kovacic & Somanathan 2014) and known to trigger and maintain catalytic reaction cycles causing oxidative stress and inflammation at the cellular level (Bolton et al. 2000; Andersson et al. 2009; Song & Buettner 2010). Antioxidants in epithelial lining fluid (ELF) to redox-cycle quinones (Roginsky et al. 1999; Kelly et al. 2018). The redox-active species produce reactive oxygen species (ROS) in the ELF of the respiratory tract (Charrier and Anastasio 2011; Lakey et al. 2016). ROS cause oxidative stress associated with chronic diseases (Sarnat et al. 2008; Krall et al. 2017).

PACs are emitted by combustion of fossil fuel and biomass or can be formed by photochemistry. They are found in urban aerosols (Albinet et al. 2008; Lammel 2015; Shen et al. 2016), but also in the continental background and at remote sites (Lammel et al. 2017; Nežiková et al. 2021; Wietzoreck et al. 2022) in both gaseous and particulate phases.

The potential to cause oxidative stress can be estimated utilizing cellular or acellular oxidative potential (OP) assays, which quantify ROS formation or antioxidant consumption. Hereby, PM is added to an assay reagent either directly or as an extract (bulk mixing; Ayres et al. 2008; Verma et al. 2015; Crobeddu et al. 2017;Calas et al. 2018; Daellenbach et al. 2020) or as air–liquid interface (ALI) cell exposure system (Paur et al. 2011; Endes et al. 2014).

Redox-active components of PM_2.5_ can become effective in the lung through the following mechanisms: (i) molecular dissolution in ELF, (ii) deposition of inhaled particles to alveolar or tracheobronchial surfaces covered by ELF, or (iii) carriage by ultrafine particles (UFPs) penetrating the lung epithelia (Geiser et al. 2005; Li et al. 2017). The surface area presented by the alveoli of the human lung is large (≈100 m^2^) relative to the volume (≈0.35 L, adult; USEPA 2011, 2019), resulting in alveolar-specific surface area to volume of ≈285 m^2^ L^−1^ (Weibel 1963). The ELF-covered surface area could play an important role in the phase transfer and/or the efficiency of catalytic activity of redox-active components of UFPs (Borm et al. 2006; Hussain et al. 2009), i.e. mechanism (ii), which has not been explored yet. Many redox-active species are insoluble in water and thus difficult to disperse in aqueous solution. To our knowledge, no method has been described to load poorly water-soluble organics into ELF. Batch experiments with the target substances provided in bulk solution or as solids may cover mechanism (i) but are expected to largely underestimate mechanism (ii) and completely neglect (iii). Furthermore, organic solvents needed to mimic (ii) may be incompatible with cellular and even acellular biotests (Ayres et al. 2008).

This study aimed to (a) design a method to load poorly water-soluble redox-active organics abundant in ambient fine PM into ELF via an ALI area and (b) determine the OP of this loaded ELF using off-line acellular assays. To this end, an aerosol of UFPs, loaded with the target compounds, is scrubbed by a simulated ELF nebulized in a mist chamber providing the large interface area. Hereby, we test the hypothesis that a high target substance concentration in ELF can be achieved, higher than by bulk mixing. The OP of ELF loaded via ALI mixing is compared with the OP of the same amounts of redox-active substances in ELF following bulk mixing.

## Methodology

### Choice of ELF

The ELF chosen is a widely used modified Gamble’s solution (Boisa et al. 2014; composition see Table S1) which accounts for realistic levels of electrolytes, antioxidants (namely ascorbic acid, uric acid, and glutathione), proteins, and surfactants. The preparation of the ELF accounting for various requirements is explained in the supplementary material (SM), S1.2.

### ELF loading with redox-active organics

#### ELF loaded by scrubbing nano-particles coated with redox-active organics

**Fig. 1:**
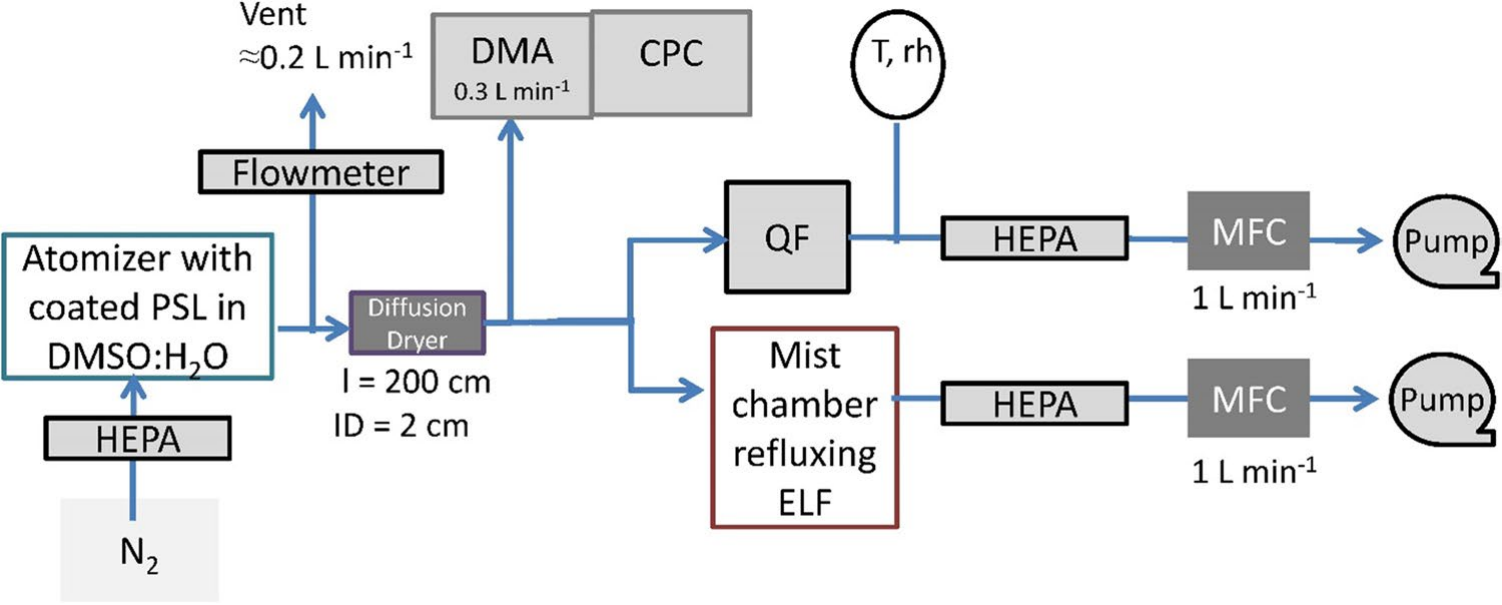
Schematic of experimental setup (CPC, condensation particle counter; DMA, differential mobility analyser; HEPA, high-efficiency particulate air filter; MFC, mass flow controller; PSL, polystyrene latex particles; QF, quartz fibre filter; rh, relative humidity; T, temperature)

Dry coated UFPs were produced by an atomizer and downstream diffusion dryer (Fig. 1). Un-modified, hydrophobic polystyrene latex (PSL) spheres were used in an 80:20 mixture of dimethyl sulfoxide (DMSO) and ultrapure water. PSL provide an inert and suitable matrix for delivery of hydrophobic substances (Lieberherr et al. 2021). The performance characteristics of the atomizer are detailed in the SM, S1.2, and Fig. S1. The targeted organics were added to the 80:20 DMSO:H_2_O solution containing ≈0.1% PSL spheres with a mean diameter of 57 nm (5%_w_ polystyrene, 1.05 g cm^−3^_,_ < 0.1%_w_ surfactant tenside solution PS055LT from ConSenxus, Ober-Hilbersheim, Germany). Particles were then atomized by a slightly modified atomizer (model 3076 Constant Output Atomizer, TSI, Shoreview, USA) under stirring agitation with particle-free N_2_. The 2–4 ring quinones and nitrated PAHs (NPAHs) were targeted based on their abundance in ambient air as well as their expected OP (listed in Table S2). In the downstream dryer, the organic solvent was completely removed due to evaporation. The repeated precision of the generated UFPs with mode around 60 nm is illustrated in Fig. S1.

During operation, the TSI atomizer in combination with the dryer effectively generated ≈4.5 × 10^−3^ m^2^ s^−1^ of total surface area for distributions shown in Fig. S1. The scanning mobility particle sizer (SMPS) consisted of a differential mobility analyser (DMA) and a condensation particle counter (CPC model 5416, Grimm, Ainring, Germany). This aerosol was scrubbed in a mist chamber, model BF61400 Aero Mist nebulizer (Allied Healthcare Products, St. Louis, USA), originally designed for application of aerosolized drugs. It is modified from Cofer et al. (1985), operates on the Venturi principle, and can nebulize up to 15-mL liquid. Supersaturation conditions had been successfully used for efficient scrubbing of aerosols earlier (Orsini et al. 2008). In this chamber, in a volume of ≈15 mL, a mist of the extracting solution (ELF) is nebulized, impinged on a hydrophobic membrane (Teflon filter), and recycled at a rate of ≈0.5 mL min^−1^ with a total droplet surface area of ≈10^−7^ m^2^ s^−1^ (extrapolated to ≈1 L min^−1^ sample aerosol flow from Fig. S4). Considering scrubbing periods of 20–40 min, the total effective ELF surface area provided for reactive uptake was (1.5 ± 0.5) × 10^−5^ m^2^ L^−1^.

The generated particles are deposited onto the quartz fibre filter (QF) in one channel and are scavenged by refluxed ELF (15 mL, PTFE filter) in a mist chamber. The mass flux of target substances from the atomizer is quantified by analysis of the deposit on the QF. To this end, the filter was extracted with dichloromethane (DCM), and the extract was purified with solid-phase extraction using a SiOH cartridge and then quantified (Chemical analysis).

The interfacial surface area produced in the mist chamber at 1 L min^−1^ was 0.4 ± 0.1 m^2^ min^−1^, which yielded an operation time dependent total interface area between 8 and 17 m^2^ for the given test run times between 20 and 40 min (Table 1). Note that the lung of an adult human averages 100 m^2^ (Weibel 1963).

**Table 1:** Concentration and OPs of ELF samples loaded with 1–2 quinones or NPAHs following air–liquid interface (ALI) or bulk mixing (last column). OP values represent the mean of 2 (OP^DTT^) or 3 (OP^AA^, OP^H2O2^) replicates each ± standard deviation

Redox-active substance(s)	Concentration(s) (µg L^−1^)	Interface area (m^2^)	OP^DTT^ (µM min^−1^)	OP^AA^ (µM min^−1^)	OP^H2O2^ (µM)	OP^H2O2^ (µM)
		Air–liquid interface mixing				Bulk mixing
1,4-O_2_NAP	< 0.05 0.1 0.2	8.3 12.5 16.6	- - 0.16 ± 0.02	0.033 ± 0.031 < 0.045 0.051 ± 0.036	< 0.030 0.022 ± 0.009 0.12 ± 0.07	
2 M-(1,4)O_2_NAP	< 5^a^	16.6	-	0.065 ± 0.037	0.088 ± 0.005	
9,10-O_2_ANT	317 793	8.3 16.6	- -	< 0.036 -	- 0.013 ± 0.006	- < 0.10
9,10-O_2_PHE	187^a^	16.6	-	0.45 ± 0.04	0.26 ± 0.01	
7,12-O_2_BAA	256 856	8.3 16.6	< 0.02 -	< 0.030﻿–	0.11 ± 0.01﻿–	< 0.04﻿–
1-NNAP	86	12.5	-	0.040 ± 0.027	< 0.05	< 0.04
9-NPHE	211	12.5	-	0.035 ± 0.033	< 0.05	< 0.04
1-NPYR	511	12.5	-	0.054 ± 0.029	0.17 ± 0.05	< 0.04
1,4-O_2_NAP + 1-NNAP	< 0.05 + 55	12.5	0.24 ± 0.05		0.09 ± 0.02	
1,4-O_2_NAP + 9-NPHE	< 0.05 + 312	12.5	0.17 ± 0.04		0.05 ± 0.01	
1,4-O_2_NAP + 1-NPYR	< 0.05 + 218	12.5	0.20 ± 0.04		0.22 ± 0.02	
1,4-O_2_NAP + 9,10-O_2_PHE	98 + 182^a^	16.6	4.18 ± 0.27		9.61 ± 0.3	

^a^Concentration value with higher uncertainty

In order to preserve the target compounds, the ELF used for scavenging was employed with a modified composition; namely antioxidants (ascorbic acid, uric acid, and glutathione) as well as cysteine were not included. The reason being antioxidants might modify the redox-active substances, while cysteine bears the risk of unwanted reactions with amino acid groups during storage. Storage temperature was 4 °C. Right before OP measurements, the antioxidants ascorbic and uric acid were added to the loaded ELF solution. OP measurements were done using the H_2_O_2_ assay (OP^H2O2^; MAK-165, Sigma-Aldrich 2014), antioxidant assay (OP^AA^; Shahpoury et al. 2019), and the DTT assay (OP^DTT^; Tong et al. 2018), described in the SM. These acellular methods are the most frequent choices to determine the OP of organics and especially quinones (Guo et al. 2020). The H_2_O_2_ assay presents the physiological conditions best, since it includes the endogenous antioxidant in physiologically relevant concentrations in a simulated ELF measuring a product, which is also produced in the human body. Acellular OP assays including these have recently been intercompared (Shahpoury et al. 2022). Samples from the QF and the mist chamber were blank controlled (producing daily minimum of one ELF and one QF blank, which remained unloaded but otherwise followed the exact same lab treatment and handling as the loaded ELF and QF samples, respectively). At the beginning of each day’s experiments, the atomizer was flushed first with a blank 80:20 DMSO:H_2_O solution.

#### ELF loaded by bulk mixing of redox-active organics

The identical concentrations of redox-active substances in bulk mixtures in ELF as in the samples of scrubbing ELF were produced in a beaker. For at least some of the compounds, these concentrations (100–1000 µg L^−1^; Table 1) were limited by the substances’ solubility in ELF. The concentrations were achieved in several dilution steps, assuring the organic solvent not to exceed organic:H_2_O of 1:100. Two microlitres of the respective PAC stock solution in DMSO (0.1 g L^−1^, then diluted to the tenfold targeted concentration) with the respective concentration was added to 198-µL ELF and vortexed (2 min). The OP^H2O2^ was determined subsequently (S1.4.1) after adding the antioxidants ascorbic and uric acid.

### Chemical analysis

The quartz filter samples were extracted by vortexing with dichloromethane (DCM) and then cleaned-up using solid-phase extraction (SPE, SiOH cartridge, Macherey Nagel, Düren, Germany; Albinet et al. 2014). The target substances were isolated from ELF scrubber samples by applying an aliquot (0.5, 1, or 3 mL depending on the substance) by protein precipitation (in a centrifuge vial with equivalent volume of isopropanol, vortexing and centrifugation) and SPE (HLB cartridge, Macherey Nagel). Because of low recovery of SPE in case of 1,4-naphthoquinone (1,4-O_2_NAP), 2-methyl-1,4-naphthoquinone (2 M-(1,4)O_2_NAP), and 9,10-phenanthrenequinone (9,10-O_2_PHE), liquid–liquid extraction using DCM was performed with subsequent purification by SPE (SiOH cartridge) for these three quinones. The analytes were separated and quantified using a Trace 1310 gas chromatograph (GC; Thermo Scientific, Waltham, USA) interfaced to a TSQ8000 Evo triple quadrupole mass selective detector (MS/MS; Thermo Scientific). The analysis was performed in negative chemical ionization mode with methane used as the ionization gas. The analytes were separated on a 30 m DB-5 ms capillary column (0.25 mm ID, 0.25 μm film thickness; J&W, Santa Clara, USA) with helium (99.99%; Westfalen, Münster, Germany) as a carrier gas (Shahpoury et al. 2018). Analyte recoveries (Table S2) from ELF and QFs ranged 85–116% (*n* = 4 replicates).

## Results and discussion

### ELF loading by air–liquid interface and related OP

The concentration of the target substances in the scrubbed ELF increased with the loading time (i.e. collection time of mist chamber, Table S3) and was proportional to the amounts of loaded compounds. However, a trend for volatilizational losses from the loaded PSL was found in the QF samples, suggesting that low vapour pressure (high molecular weight or MW) substances were enriched, as high vapour pressure substances likely partitioned to some extent to the gas-phase (gas-particle partitioning). The amounts of redox-active substances loaded into ELF by scrubbing were similar to those collected on the filter for the high MW (low vapour pressure) compounds (relative difference between 50 and 300%) but were higher for the low MW (high vapour pressure) compounds (relative difference exceeding 1 order of magnitude; Table S3). This points to the advantage of using mist chamber for scavenging of the gas-phase in equilibrium with suspended particles by the scrubbing ELF. This is a more realistic representation of the uptake into ELF in the lung than using particles’ deposits, collected by phase separation.

### Comparison of air–liquid interface (ALI) mixing with bulk mixing

For two substances tested, 7,12-O_2_BAA and 1-NPYR, a significant OP^H2O2^ is found following uptake across the large ALI, while no OP was detected for the same (stoichiometric) mixture produced by bulk mixing of the redox-active compounds into ELF (Table 1). For one other quinone, 9,10-O_2_ANT, and two other NPAHs, 1-NNAP and 9-NPHE, no or very low OP was found regardless of the type of mixing or uptake (Table 1). The observed effect reflects a more efficient phase transfer in the mist compared to the bulk, validating the hypothesis. While bulk mixing bears impediments of agglomeration, insolubility, and poor miscibility, the scrubbing process provides a large surfactant surface area for molecules at the PSL particles’ surface to adsorb on and become available and accessible for reaction later on. The water solubility of the studied 2-ring substances is high (> 1 g L^−1^), but of the 3–4 ring substances limited (0.3–54 mg L^−1^). 1-NPYR is known to get redox cycled by proteins (present in ELF; Table S1; Nachtman 1986; Park & Park 2009), which was not reported from the other studied NPAHs. A surface effect on redox activity has not been reported for quinones or other oxidized aromatics yet, to the best of our knowledge. However, the decomposition of gaseous ozone on water droplets (and related formation of H_2_O_2_; Gallo et al. 2022) was found to be mediated by the interface surface area. Possibly, a reaction could be facilitated, if the condensed phase matrix enhanced the exposure of one of the reactants, through reducing degree of freedom of molecules and/or accumulation at the interface. This was reported for the reactivity of an aromatic alkene on the surface of ice (Ray et al. 2013).

Apart from interface area in the alveolar space and water solubility, the efficiency of the uptake of particulate phase redox-active compounds into ELF in the lung will also depend on PM matrix (wettability, morphology) and on the distribution of redox-active compounds in the particles (surface, bulk). This is suggested by leaching PM samples from various sources and of different morphology with simulated ELF (Xie et al. 2018; Liu et al. 2019). Up to date, such aerosol parameters’ effects have not been specifically addressed and were beyond the scope of this study.

## Conclusions

Through reproducible generation of nano-particles coated with redox-active compounds, a large surface area for uptake of these compounds into ELF was provided. The mist chamber allows for both uptake mechanisms following dissolution as well as surface-mediated multiphase reactions at the ELF-nano-particle interface and provides soft deposition conditions. OP quantified by application of a mist chamber also includes the gas-phase of semi-volatile components of PM, which is more realistic to the actual uptake in the lung than quantification based on filter deposits, whose constituents had been separated from the gas-phase.

Among the 5 substances comparing effects from ALI mixing with bulk mixing, a significant OP signal (*t*-test, 0.05 level of significance) was found for two oxidants (7,12-O_2_BAA and 1-NPYR) when the ELF uptake occurred via a large ALI, while all 5 compounds yielded negligible OP signal when mixed in bulk. This suggests that the OP of ambient PM as determined in acellular assays by bulk mixing of extracts of filter or impactor samples might be lower than the effective OP exerted in the lung upon inhalation. The specific surface concentration of the experimental setup used, 8 m^2^ during 20 min or 0.03 m^2^ per 5 s (average duration of breath), still corresponds to 3 orders of magnitude which reduced area relative to the conditions in the deep lung, i.e. ≈10^2^ m^2^ exposed every 5 s. Hence, the effect of redox-active components of ambient PM might be even stronger in the deep lung, and the effective OP is correspondingly higher. Our results indicate that the OP of PM components depends not only on the PM substance properties but also on the ELF interface properties and uptake pathways. OP measurements based on bulk mixing of phases, for which data have been accumulating rapidly in recent years, may not represent the effective OP in the human lung with its large interfacial area.

## Supplementary Information

Below is the link to the electronic supplementary material.

Supplementary file1 (PDF 396 KB)

## Data Availability

All data generated or analysed during this study are included in this published article, supplied as supplementary material, or are available from the corresponding author on reasonable request.
