# Supplementary material for: Is the oxidative potential of components of fine particulate matter surface-mediated?
Source: Environ Sci Pollut Res Int. 2022 Dec 23;30(6):16749–55. doi: 10.1007/s11356-022-24897-3 (PMC9908692; doi:10.1007/s11356-022-24897-3)
Supplement: Supplementary file 1 — Supplementary file1 (PDF 396 KB) [file 11356_2022_24897_MOESM1_ESM.pdf]

Supplementary material

Oxidative potential of components of fine particulate matter is surface-mediated

Baumann K.[1,2,3], Wietzoreck M. [1], Shahpoury P. [1,4,5], Filippi A. [1], Hildmann S.[1], Lelieveld S. [1], Berkemeier T. [1], Tong H. [1,6], Pöschl U. [1], Lammel G. [1,7]

[1] Max Planck Institute for Chemistry, Multiphase Chemistry Department, Mainz, Germany
[2] University of North Carolina, Department of Environmental Sciences and Engineering, Chapel Hill, USA
[3] Picarro Inc., Santa Clara, USA
[4] Environment and Climate Change Canada, Air Quality Processes Research Section, Toronto, Canada
[5] Trent University, Chemistry Dept., Peterborough, Canada
[6] Helmholtz-Zentrum Hereon, Institute of Surface Science, Geesthacht, Germany
[7] Masaryk University, Research Centre for Toxic Compounds in the Environment, Brno, Czech Republic

S1. Methods

S1.1 Epithelial lining fluid

Table S1. Composition of the epithelial lung lining fluid (ELF; mg L^{-1}) used i.e., modified Gamble's solution following *Boisa et al., 2014.*

Substance	Concentration in ELF
NaCl	6020
$CaCl_2$	256
Na_2HPO4	150
$NaHCO_3$	2600
KCl	298
$MgCl_2$	200
Na_2SO_4	72
Ascorbic acid (AA) 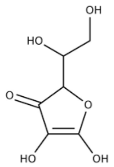	18
Uric acid (UA) 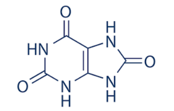	16
Glutathione	30
Albumine	260
Dipalmitoylphosphatidylcholine (DPPC)	100
Glycine H_2N-CH_2-COOH	376

pH	7.4

Deviating from *Boisa et al., 2014*, mucin was excluded for the following consideration: Bacterial impurities have proven to influence the catalytic oxidation of the red peroxidase substrate in the H_2O_2 assay. Using Hela TLR4 dual reporter cells in an intracellular test revealed synergistic mechanisms in multiplying the available endotoxin content. This induced a strong TLR4 stimulation, suggesting a highly inflammatory potential of the ELF. Impurities in the form of bacterial lipid polysaccharides (LPS) introduced by the protein ingredient mucin are suspected to be the culprit for this artefact reaction.

The limited solubility of DPPC could be solved based on a method proposed by *Abate et al. (2010)*: DPPC was dissolved in DCM/methanol 1:1 (v:v) using a glass beaker. Subsequently, the solvent was evaporated in the fume hood on a heating plate with approx. 60°C. The resulting dry film at the glass surface was re-dissolved with 200 mL of ultrapure water at 55°C and the suspension was agitated for 1-2 h. In order to obtain a homogeneous dissolution of DPPC in water, the warm suspension was sonicated.

S1.2 Organics-coated aerosol

Table S2. Target compounds selected

Analyte	Abbreviation	Structure	Water solubility (mg $L^{-1,}$ at 298 K) [a]	Comments
1,4-Naphthoquinone	1,4-O_2NAP	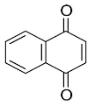	2420	Most modeled, kinetic data available, water soluble
2-Methyl-1,4-naphthoquinone (menadione, vitamin K3)	2M-(1,4)O_2NAP	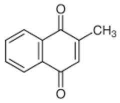	794	Abundant, kinetic data available
9,10-Anthraquinone	9,10-O_2ANT	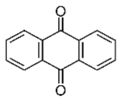	3.92	Most abundant in UFP fraction
9,10-Phenanthrenequinone	9,10-O_2PHE	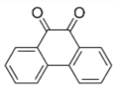	21.7	Abundant and much studied experimentally
Benz[a]anthracene-7,12-dione	7,12-O_2BAA	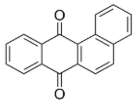	0.29	Abundant and much studied experimentally

1-Nitronaphthalene	1-NNAP	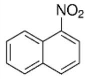	12500	Co-emitted with most quinones
9-Nitrophenanthrene	9-NPHE	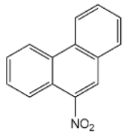	53.5	Highly toxic NPAH
1-Nitropyrene	1-NPYR	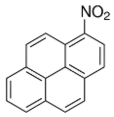	12.5	Kinetic data available, most studied and highly toxic NPAH

[a] estimated *(USEPA, 2012)*

The particle mode with highest number concentration at 57 nm reflects the uncoated PSL particles (mean nominal size of substance PS055LT40 from ConSenxus GmbH, Ober-Hilbersheim, Germany). Larger particles reflect agglomeration products. Identical atomizer operation with same PSL concentration in the 80:20 DMSO:ultrapure water solution (dynamic blank) yields a similar size distribution at smaller number concentration (see black trace). The occurrence of the broad profile must be an effect of impurities in the manufactured PSL or in the lab water, which serves as baseline profile for the NOPAH loaded distribution; i.e. the net NOPAH mass detected by the SMPS is determined by the difference of the two different distributions.

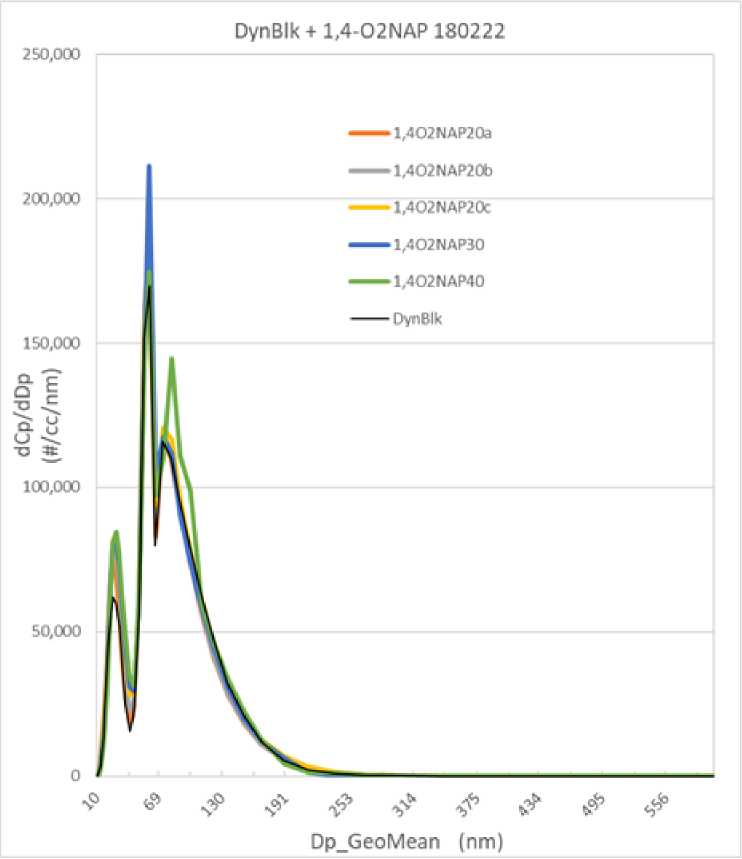

Fig. S1: Reproducible aerosol mass size distribution of PSL particles coated with 1,4-naphthoquinone and as dynamic blank (DynBlk).

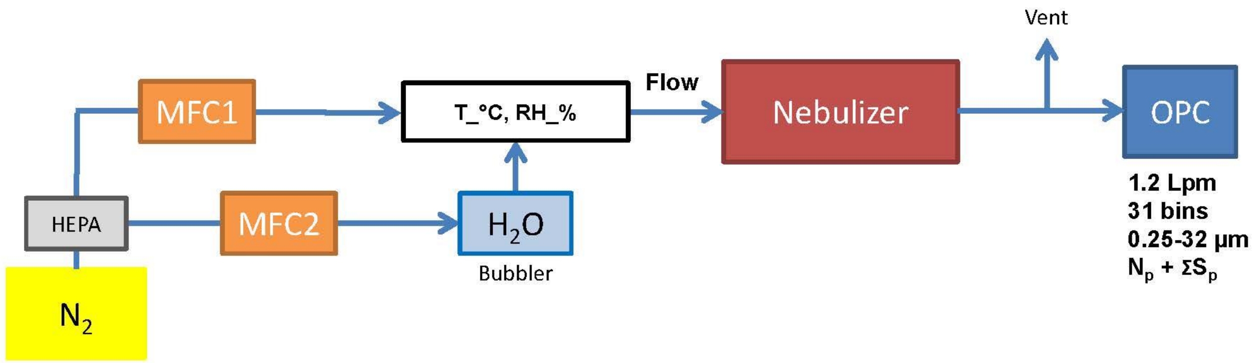

Fig. S2: Test set up to determine droplet number and surface distribution achieved by the mist chamber nebulizing the ELF medium as function of flow rate. HEPA = high efficiency particulate air filter, MFC = mass flow controller, OPC = optical particle counter

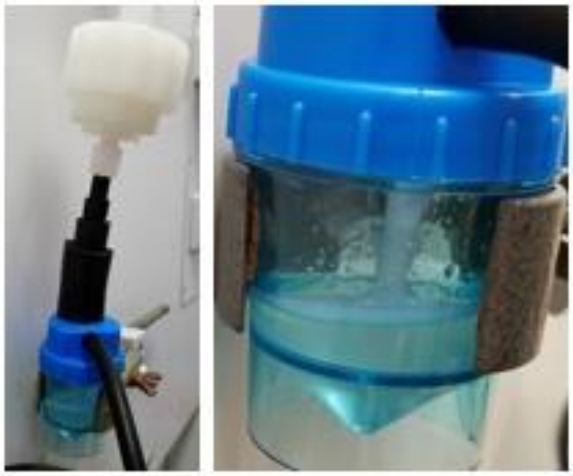

Fig. S3: Nebulizing mist chamber with 0.2 um Teflon filter membrane (Zefluor 47 mm PTFE, Pall Laboratory) inside holder on top of outlet (left), causing ELF to constantly reflux into solution at bottom well (right).

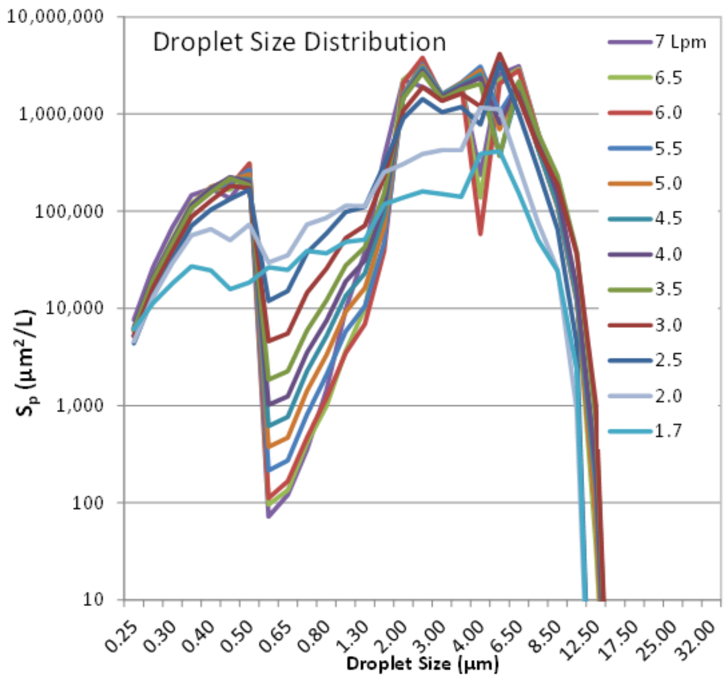

10/31/17 From/To	Flow Lpm	N_p #/L	ΣS_p µm²/L	ΣS_p µm²/min
14:38:12	**1.7**	478,165	2,088,442	3,550,352
14:39:30	*RSD*	*9.5%*	*15.9%*	*15.9%*
14:40:48	**2.0**	972,594	5,213,151	10,426,302
14:42:06	*RSD*	*2.6%*	*3.6%*	*3.6%*
14:43:18	**2.5**	1,444,214	11,156,553	27,891,384
14:44:36	*RSD*	*2.1%*	*7.1%*	*7.1%*
14:45:48	**3.0**	1,669,413	14,504,335	43,513,005
14:47:06	*RSD*	*2.0%*	*3.9%*	*3.9%*
14:48:06	**3.5**	1,907,099	13,817,099	48,359,846
14:49:24	*RSD*	*2.2%*	*1.1%*	*1.1%*
14:50:36	**4.0**	2,007,964	14,548,030	58,192,120
14:51:54	*RSD*	*2.8%*	*6.3%*	*6.3%*
14:52:48	**4.5**	2,021,329	14,445,268	65,003,706
14:54:06	*RSD*	*2.4%*	*3.0%*	*3.0%*
14:55:06	**5.0**	2,064,478	15,383,633	76,918,163
14:56:24	*RSD*	*2.0%*	*2.7%*	*2.7%*
14:58:18	**5.5**	2,037,401	16,243,160	89,337,379
14:59:36	*RSD*	*2.3%*	*2.8%*	*2.8%*
15:00:06	**6.0**	2,090,574	16,109,736	96,658,417
15:01:24	*RSD*	*3.1%*	*2.0%*	*2.0%*
15:02:36	**6.5**	2,132,600	16,180,141	105,170,918
15:03:54	*RSD*	*2.9%*	*3.9%*	*3.9%*
15:04:36	**7.0**	2,141,905	15,639,959	109,479,716
15:05:12	*RSD*	*2.2%*	*1.8%*	*1.8%*

Fig. S4: ELF droplet surface size distribution generated by the nebulizing mist chamber (left) and as sum totals of number and surface concentrations (right) for different flow rates. The optical particle counter used is Grimm model 1.109, 31 channels; Grimm, Ainring, Germany.

S1.4 Acellular OP assays

S1.4.1 H_2O_2 formation assay

The H_2O_2 assay measures the formation of H_2O_2 in solution (or dispersion). We used the commercially available fluorescence-based hydrogen peroxide assay kit (MAK165, Sigma Aldrich, Schnelldorf, Germany; *Sigma-Aldrich, 2014*). The formed H_2O_2 is determined by the reaction with non-fluorescent red peroxidase substrate Amplite™ ADHP, chemically similar to Amplex Red (10-acetyl-3,7-dihydroxyphenoxazine), using horseradish peroxidase (HRP) as catalyst to form highly fluorescent resorufin. The assay was performed following *Tong et al. (2018)*: The two antioxidants ascorbic acid and uric acid were added prior to the start of the experiment. Stock solutions of uric acid (Sigma Aldrich) (3.2 g L^{-1}) in 0.05 M NaOH (Sigma Aldrich) and ascorbic acid (Sigma Aldrich) (1.8 g L^{-1}) in ultra-pure water were prepared in Nalgene HDPE bottles (Fisher Scientific, Schwerte, Germany) to prevent contamination by transitions metals from glass surfaces. The mixture was gently shaken after the addition and subsequently incubated for 20 min at room temperature without shaking. Afterwards, the H_2O_2 detection reagent was added according to the assay instructions. After additional 15 min, the fluorescence (540 nm excitation and 590 nm emission) was measured using a plate reader (Synergy NEO, BioTek Instruments, Winooski, USA). In all samples, the final H_2O_2 concentration was

estimated using a calibration based on 0-100 µM H_2O_2 in phosphate buffer. Each mixture of analytes was measured in triplicate samples.

S1.4.2 DTT depletion assay

Dithiothreitol (DTT) is a disulfide reducing agent that has widely been used as a surrogate for reducing environments, such as the lung lining fluid. Redox-active compounds can oxidize DTT to form an intramolecular disulfide bond, and the DTT oxidation rate has been used as a proxy for oxidative potential of redox-active compounds. For the presented study, the DTT assay was performed as described in *Tong et al., 2018*. In brief, ELF loaded with the redox active substances were placed in phosphate buffered saline (pH 7.4) at a final DTT concentration of 20 µM. At indicated time intervals, the reaction was quenched using 1 mM DTNB to form a yellow reaction product (TNB^-), which was quantified using a Synergy Neo 2 multi-plate spectrophotometer at 412 nm wavelength. The calibration curve was made ranging from 0.15– 20 µM DTT. The data is presented as blank-corrected rate of DTT depletion per minute, per sample.

S1.4.3 AO depletion assay

The protocol of this assay followed *Shahpoury et al., 2019*. Briefly, 2 mL of each reference and loaded ELF samples was transferred to 8-mL reaction bottles (Nalgene, Thermo Scientific). Positive control was made by spiking 2 mL of reference ELF with 5 mg mL^{-1} suspension of standard reference particulate matter (SRM 1649b) to the final concentrations of 50 µg mL^{-1}. All samples were spiked with stock solutions of ascorbic acid and uric acid to the final concentrations of 100 µM each. 50 µL of 1 molar hydrochloric acid was added to the solution in order to adjust the pH to 7.4. The samples were incubated at 37°C in an incubator-shaker for 180 min, gently shaken. Subsequently, 300 µL of each sample was transferred to 2-mL centrifuge tubes and added with 200 µL precipitation solution containing 2% sulfosalicylic acid and 2 mM ethylenediaminetetraacetic acid, mixed for 10 s, and centrifuged at 10000 g for 6 minutes. Finally, 10 µL of the supernatant was transferred to an analytical vial containing 482 µL of 15% methanol and 8 µL of $^{13}C_6$-ascorbic acid (150 pg μL^{-1}). The samples were analyzed using an ultra-high-performance liquid chromatograph (UHPLC) coupled to a triple–quadruple mass spectrometer (MS/MS) in electrospray ionization in the negative mode. The ascorbic acid quantification was performed using the internal calibration method with six-point linear calibration curve ($r^2 = 0.999$) ranging from 1 to 250 pg μL^{-1}. Ascorbic acid depletion rate (µM min^{-1}) was calculated as follows: ($AA_{REF} - AA_{PSL}$)/(incubation time), where AA_{REF} and AA_{PSL} are the

measured concentrations (µM) of ascorbic acid in the reference and the loaded ELF sample, respectively, after 180 min. The uncertainty of results was determined to be ±0.023 µM min^{-1}.

S2. Loading experiments scrubbing nano-particles coated with redox-active organics

Table S3: Mean target substance mass concentrations in ELF (1-3 replicates) and the amount deposited on the filter (QFF).

Sample name	Loading time [min]	Substance	Concentration in ELF [ng mL^{-1}]	Amount on filter [ng]
1,4-O_2NAP	20	1,4-O_2NAP	<0.05	0.27
	30	1,4-O_2NAP	0.1	0.054
	40	1,4-O_2NAP	0.2	4.23
2-M-1,4-O_2NAP	20	2-M-1,4-O_2NAP	<5	57.1
	40	2-M-1,4-O_2NAP	-	501
9,10-O_2ANT	20	9,10-O_2ANT	317	17100
	40	9,10-O_2ANT	793	34600
9,10-O_2ANT + 1,4-O_2NAP	20	1,4-O_2NAP	0.5	229
		9,10-O_2ANT	466	17200
	40	1,4-O_2NAP	0.5[a]	298
		9,10-O_2ANT	770[a]	987000[b]
9,10-O_2PHE	20	9,10-O_2PHE	23 [a]	6810
	40	9,10-O_2PHE	187 [a]	12000
9,10-O_2PHE + 1,4-O_2NAP	20	1,4-O_2NAP	<0.05	22.1
		9,10-O_2PHE	30	9310
	40	1,4-O_2NAP	98	256
		9,10-O_2PHE	182 [a]	4830
7,12-O_2BAA	20	7,12-O_2BAA	256	33800[b]
	40	7,12-O_2BAA	856	34000[b]
9-NPHE	30	9-NPHE	211	13500
9-NPHE + 1,4-O_2NAP	30	1,4-O_2NAP	<0.05	0.78
		9-NPHE	312	24300[b]
1-NPYR	30	1-NPYR	511	15600
1-NPYR + 1,4-O_2NAP	30	1,4-O_2NAP	<0.05	1.09
		1-NPYR	218	4750
1-NNAP	30	1-NNAP	86	48.5
1-NNAP + 1,4-O_2NAP	30	1,4-O_2NAP	<0.05	1.20
		1-NNAP	55	79.1

[a] concentration with high uncertainty

[b] concentration with high uncertainty since concentration exceeded calibration range

Supplementary references

Abate W, Alghaithy AA, Parton J, Jones KP, Jackson SK (2010) Surfactant lipids regulate LPS-induced interleukin-8 production in A549 lung epithelial cells by inhibiting translocation of TLR4 into lipid raft domains. *J. Lipid Res. 51*, 334-344

Boisa N, Elom N, Dean JR, Deary M.E., Bird G, Entwistle JA (2014) Development and application of an inhalation bioaccessibility method (IBM) for lead in the PM_{10} size fraction of soil, *Environ. Int. 70*, 132-142

Shahpoury P, Harner T, Lammel G, Leliveld S, Tong H, Wilson J (2019) Development of an antioxidant assay to study oxidative potential of airborne particulate matter, *Atmos. Meas. Techn. 12*, 6529–65399

Sigma-Aldrich, Fluorimetric Hydrogen Peroxide Assay Kit MAK165, *Sigma Aldrich Tech. Bull.*

Tong H, Lakey PSJ, Arangio AM, Socorro J, Shen F, Lucas K, Brune WH, Pöschl U, Shiraiwa M (2018) Reactive oxygen species formed by secondary organic aerosols in water and surrogate lung fluid, *Environ. Sci. Technol. 52*, 11642-11651

USEPA (2012) Estimation Programs Interface Suite™ for Microsoft® Windows, v4.11. United States Environmental Protection Agency, Washington, USA
